# Post-translational modification in hepatocellular carcinoma resistance: molecular mechanisms and therapeutic targeting

**DOI:** 10.3389/fimmu.2026.1914652

**Published:** 2026-09-02

**Authors:** Simin Feng, Jie Hu, Xiaoxi Guo, Ziyi Xu, Hailiang Wang, Huanzhang Niu, Xinyang Li

**Affiliations:** 1 Department of Interventional Radiology, The First Affiliated Hospital, College of Clinical Medicine, Henan University of Science and Technology, Luoyang, China; 2 Department of Anesthesiology, Luoyang Central Hospital, Luoyang, China; 3 Henan Key Laboratory of Cancer Epigenetics, Cancer Institute, The First Affiliated Hospital, College of Clinical Medicine, Henan University of Science and Technology, Luoyang, China; 4 Department of Gastrointestinal Surgery, People’s Hospital of Zhengzhou University, Henan Provincial People’s Hospital, Zhengzhou, China

**Keywords:** hepatocellular carcinoma, immunotherapy, post-translational modifications, targeted therapy, therapeutic resistance

## Abstract

Hepatocellular carcinoma (HCC), a malignancy with high global morbidity and mortality, has therapeutic resistance as a major focus of both clinical and basic research. In recent years, protein post-translational modifications (PTMs), key regulators of intracellular protein function and cell signaling, play critical roles in therapeutic resistance in liver cancer. Current studies indicate that diverse PTMs—including phosphorylation, ubiquitination, acetylation, lactylation, and glycosylation—modulate liver cancer cell responses to chemotherapy, targeted therapy, immunotherapy and local therapies. This review systematically summarizes the molecular mechanisms and common pathways through which PTMs contribute to therapeutic resistance in liver cancer, explores PTM-mediated mechanism underlying tumor persistence and distant recurrence,examines how PTM-targeted strategies may reverse resistance, and discusses the challenges and prospects of these therapeutic approaches in clinical practice. By integrating current research, this review aims to provide a theoretical basis and potential targets for precision therapy of drug-resistant liver cancer, offering novel intervention targets and translational strategies to overcome therapeutic resistance in HCC.

## 1 Introduction

Hepatocellular carcinoma (HCC), one of the most common malignancies worldwide, accounts for more than 85% of all primary liver cancers (1). Its mortality ranks third globally and second in China (2, 3). Although surgical resection remains the preferred treatment for early-stage HCC, more than 60% of patients in China are diagnosed at an intermediate or advanced stage owing to the insidious onset of the disease, thereby missing the opportunity for curative surgery (2). For these patients, systemic therapies centered on tyrosine kinase inhibitors (TKIs) and immune checkpoint inhibitors (ICIs) have become the main treatment options. However, the rapid emergence of acquired resistance has become a major bottleneck in improving the clinical outcomes of patients with advanced HCC (2, 4, 5).

The development of drug resistance in HCC is not driven by a single factor but results from multidimensional complex mechanisms such as tumor microenvironment remodeling, immune escape, and metabolic reprogramming (6–9). In this regulatory network, protein post-translational modifications (PTMs) act as molecular switches (10). From phosphorylation, ubiquitination, acetylation, glycosylation to the emerging lactylation, PTMs can rapidly regulate protein activity, stability, and subcellular localization without altering the gene sequence (11). They not only directly promote tumor cell proliferation and invasion but also profoundly influence therapeutic sensitivity. For example, glycosylation- and ubiquitination-mediated regulation of PD-L1 immunosuppressive function can significantly affect responses to immunotherapy, whereas dysregulation of deubiquitinating enzymes (DUBs) may result in excessive accumulation of oncogenic proteins, thereby inducing resistance to targeted therapies (12, 13).

Therefore, therapeutic resistance can no longer be fully explained solely at the genetic level, and elucidating the dynamic interplay between PTMs and treatment resistance has become a pressing clinical and scientific challenge. In-depth investigation of this core regulatory network may help uncover the fundamental regulatory basis of tumor drug resistance and provide a theoretical foundation for the development of novel therapeutic sensitization strategies. It is noteworthy that the initiation and progression of HCC are profoundly shaped by diverse etiological backgrounds. Chronic HBV/HCV infection and metabolic disorder–associated factors can differentially remodel tumor metabolic landscapes and immune ecosystems, thereby conferring distinct context-dependent features upon PTM regulatory networks. Incorporating etiological heterogeneity into the analysis of PTM-driven therapeutic resistance may therefore enhance the translational relevance and broaden the applicability of precision oncology strategies.In this review, we systematically summarize the specific molecular mechanisms by which PTMs contribute to resistance to chemotherapy, targeted therapy, immunotherapy, and locoregional therapies in HCC. We then delineate shared PTM-mediated mechanisms underlying tumor persistence and distant recurrence, including key processes such as subclonal evolution and cancer stem cell (CSC) maintenance. Finally, we discuss therapeutic strategies targeting PTM-related “writers,” “erasers,” and “readers,” as well as their potential for clinical translation, with the aim of providing new insights into improving the clinical outcomes of patients with HCC.

## 2 Protein post-translational modifications and chemoresistance in HCC

### 2.1 Regulatory mechanisms of PTMs in oxaliplatin resistance

Oxaliplatin, a key component of the FOLFOX4 chemotherapy regimen, induces tumor cell apoptosis by forming platinum-DNA adducts and represents an important therapeutic option for advanced hepatocellular carcinoma (14). However, cancer cells develop complex resistance mechanisms through multidimensional PTM networks.

Among these mechanisms, mitochondrial metabolic regulation has attracted considerable attention in recent years.Adenylate kinase 2 (AK2) is a mitochondrial enzyme involved in the maintenance of cellular nucleotide homeostasis. AK2 promotes the mitochondrial localization and enzymatic activity of lysyl oxidase-like protein 3 (LOXL3) by phosphorylating LOXL3 at Ser704. Activated LOXL3 subsequently oxidizes and stabilizes dihydroorotate dehydrogenase (DHODH), a key enzyme in the de novo pyrimidine biosynthesis pathway (15). By counteracting oxaliplatin-induced ferroptosis, DHODH significantly enhances tumor cell survival under chemotherapeutic stress.

However, oxaliplatin resistance is not mediated by a single pathway, but rather arises from the coordinated involvement of multiple PTM pathways. Protein arginine methyltransferase 3 (PRMT3)-mediated arginine methylation of insulin-like growth factor 2 mRNA-binding protein 1 (IGF2BP1) reinforces pro-survival signaling, thereby facilitating tumor cell evasion of apoptosis (16). In addition, ubiquitin-specific protease 1 (USP1) promotes both deubiquitination and SUMOylation of lymphoid-specific helicase (HELLS), thereby coordinately enhancing epithelial–mesenchymal transition (EMT) and DNA damage repair capacity, which contributes to resistance to the FOLFOX regimen (17). Together, this complex network formed by the interplay among phosphorylation, methylation, and ubiquitination orchestrates drug uptake, signal transduction, and cell death programs, ultimately shaping the oxaliplatin-resistant phenotype.

### 2.2 PTMs associated with 5-fluorouracil resistance

The acquisition of 5-FU resistance largely depends on the dynamic regulation of proteostasis (18). As previously mentioned, USP1 drives EMT and DNA repair through dual modification of HELLS, also participating in 5-FU resistance (17). Similarly, ubiquitin-specific peptidase 39 (USP39) promotes cell-cycle progression and the survival of resistant cells by stabilizing structural maintenance of chromosomes 4 (SMC4) (19). Thus, ubiquitination serves as an important regulatory mechanism controlling resistance-associated proteins in this context.

However, PTM network exhibits complex functional duality during tumor progression. Ubiquitin-mediated mitophagy represents a typical example of such biphasic regulation. Yip1 domain family member 5 (YIPF5) expression can activate the stimulator of interferon genes (STING)–autophagy axis. Although basal autophagy supports tumor cell survival, agents such as salidroside can induce YIPF5 hyperactivation and mitophagic overload, thereby triggering cellular senescence and mitotic arrest and sensitizing resistant cells to chemotherapy (20).

These findings suggest that therapeutic intervention should not be confined to inhibiting resistance-promoting PTMs; rather, selective hyperactivation of specific pathways may also represent an effective strategy for reversing chemoresistance.

### 2.3 Regulatory mechanisms of PTMs in FOLFOX4 resistance

Under the FOLFOX4 combination chemotherapy regimen,HCC resistance exhibits a marked metabolic dependence, with lactylation playing a important role. Metabolic rewiring induced by chemotherapeutic stress enhances glycolytic activity in tumor cells, and the resulting lactate functions not merely as a metabolic end product but also as a key signaling molecule that drives therapeutic resistance.

This process is mediated through two coordinated pathways. First, lactate establishes a metabolic–epigenetic positive feedback loop by inducing histone H3 lysine 14 lactylation (H3K14la), which upregulates the E3 ubiquitin ligase NEDD4. Increased NEDD4 expression promotes PTEN degradation, thereby sustaining PI3K/AKT pathway activation and further enhancing glycolysis and lactate production, ultimately forming a self-reinforcing resistance circuit (21). Second, lactylation remodels the epitranscriptomic regulatory landscape. The lactyltransferase PARK7 catalyzes the lactylation of IGF2BP3, significantly enhancing its binding to and stabilization of ferroptosis suppressor protein 1 (FSP1) mRNA, thereby strengthening the capacity of tumor cells to withstand chemotherapy-induced oxidative stress and ferroptosis (22).

Thus, in the FOLFOX4-resistant model, PTMs function not only as switches in signal transduction but also as molecular links between tumor metabolic reprogramming and gene expression regulation. Targeting key lactylation events may therefore provide a potential therapeutic strategy for overcoming metabolism-dependent chemoresistance.

## 3 PTMs and resistance to targeted therapy in HCC

### 3.1 Roles of PTMs in sorafenib resistance

Sorafenib was the first approved first-line targeted therapy for advanced HCC; however, acquired resistance frequently develops, limiting its long-term clinical benefit. Mechanistic studies have shown that HCC cells exploit PTM-mediated regulatory programs to establish adaptive survival networks. These networks operate mainly through three interrelated mechanisms.

PTMs reshape metabolic flux by coordinating distinct metabolic adaptations. Lysine acetyltransferase 8 (KAT8)-mediated acetylation induces an isoform switch between phosphoenolpyruvate carboxykinase 1 and 2 (PCK1/PCK2), redirecting metabolic flux from cytosolic gluconeogenesis toward mitochondrial fatty acid oxidation and thereby establishing a reverse metabolic adaptation program (23). In parallel, S100 calcium-binding protein A14 (S100A14) suppresses ubiquitin-mediated degradation of glutaminase 1 (GLS1) by blocking its phosphorylation, thereby enhancing glutaminolysis to counteract oxidative stress (24). Together with impaired mitochondrial function mediated by the silent information regulator 1 (SIRT1)/AMPK axis, this metabolic shift drives resistant cells toward compensatory aerobic glycolysis (25). Sustained pro-survival signaling represents another major layer of PTM-mediated adaptation. Exportin 1 (XPO1)-dependent maintenance of nucleophosmin 1 (NPM1) Lys54 acetylation homeostasis cooperatively supports the resistant phenotype and EMT progression (26). Meanwhile, histone deacetylase 2 (HDAC2) stabilizes pleckstrin homology and RhoGEF domain-containing family G member 5 (PLEKHG5) through deacetylation, leading to activation of the Rac1/AKT/NF-κB signaling axis (27). A phosphorylation circuit involving integrin β5 (ITGB5) and casein kinase 1α1 (CSNK1A1) further sustains epidermal growth factor receptor (EGFR)–AKT signaling. Alongside aberrant AKT Thr308 phosphorylation, these events maintain stemness-associated properties in resistant cells (28). Meanwhile, sorafenib-induced stress activates the NOTCH1/ATM axis, triggering a phosphorylation cascade involving CHK2 and BRCA1, thereby reinforcing the resistance barrier through enhanced maintenance of genomic stability (29). Dysregulated ubiquitin-related machinery also protects resistant cells from ferroptosis. Ubiquitin-specific peptidase 22 (USP22) blocks ferroptosis by stabilizing cyclin-dependent kinase 11B (CDK11B) and transcriptionally repressing transferrin receptor 1 (TFRC) (30). Ubiquitin-specific peptidase 18 (USP18) promotes degradation of nuclear receptor coactivator 4 (NCOA4) through deISGylation, thereby suppressing ferroptosis and driving acquired resistance (31). In addition, K6-linked non-degradative ubiquitination of chaperonin-containing TCP1 subunit 3 (CCT3) at Lys21 contributes to ferroptosis inhibition (32). Furthermore, PP2A-B55β maintains the antioxidant activity of GPX4 through dephosphorylation at the Ser2 site; however, this regulatory axis is disrupted in sorafenib-resistant HCC, resulting in enhanced resistance to ferroptosis (33). Notably, the evolution of PTM-mediated therapeutic resistance may also be influenced by distinct etiological contexts. In HBV-associated HCC, miR-3677-3p suppresses the expression of the E3 ubiquitin ligase FBXO31, thereby preventing FOXM1 ubiquitination and degradation, leading to aberrant FOXM1 accumulation, reinforcement of cancer stemness, and promotion of sorafenib resistance (34). These findings suggest that virus-associated molecular landscapes may reshape PTM homeostasis and consequently modulate responses to targeted therapies.

The identification of these core targets provides a rationale for developing PTM-directed intervention strategies with translational potential. Such strategies may include pharmacological inhibition of XPO1 with KPT-8602, targeting HDAC2 to resensitize tumors to sorafenib, and combining inhibitors of USP22 and USP18, such as hyperoside. Integration of these approaches with ferroptosis inducers or PROTAC-based strategies may extend the therapeutic scope of sorafenib beyond single-agent kinase inhibition toward more systematic combination regimens.

### 3.2 PTM-mediated mechanisms of lenvatinib resistance

As a first-line therapy for HCC, the clinical benefit of lenvatinib is frequently limited by acquired resistance (35). In this process, PTMs reshape intracellular signaling and metabolic networks, influencing the fate of tumor cells (36, 37).

This remodeling is initiated at the level of plasma membrane signaling. Dolichyl-diphosphooligosaccharide—protein glycosyltransferase non-catalytic subunit (DDOST) maintains the conformational stability and aberrant activation of EGFR through glycosylation, thereby sustaining downstream prosurvival signaling (38). Meanwhile, transcriptional programs are reconfigured within the nucleus, where dephosphorylation of CREB-regulated transcription coactivator 2 (CRTC2) promotes its nuclear translocation and induces the expression of oncogenic genes (39).

The ubiquitin system further exerts precise control over protein stability. Ubiquitin-specific peptidase 15 (USP15) stabilizes galectin-3 (LGALS3) by removing ubiquitin chains, leading to activation of the AKT/mTOR pathway (40). In parallel, deltex E3 ubiquitin ligase 2 (DTX2) promotes ubiquitin-dependent degradation of t hydroxysteroid 17-beta dehydrogenase 4 (HSD17B4), thereby suppressing ferroptosis (41). Together, this bidirectional regulation of key protein stability establishes a robust resistance barrier and contributes to the maintenance of lenvatinib resistance.

Collectively, DDOST, USP15, and DTX2 represent promising therapeutic targets for overcoming lenvatinib resistance. The development of small-molecule inhibitors against these PTM regulators may disrupt upstream molecular events that drive adaptive resistance. Targeting these critical PTM regulatory nodes could therefore provide a rational strategy to restore tumor sensitivity to lenvatinib.

### 3.3 PTM-mediated signaling crosstalk and resistance network formation

During the development of resistance to targeted therapies in HCC, PTM events rarely function in isolation. Instead, they form interconnected regulatory networks that operate across multiple signaling and metabolic layers. Beyond sorafenib and lenvatinib, regorafenib resistance is similarly driven by PTM-mediated mechanisms. PRMT5-catalyzed arginine methylation stabilizes the ribosomal protein RPL14, thereby contributing to the maintenance of the resistant phenotype (42), further highlighting the pervasive role of PTM regulation in mediating resistance to TKIs. Such crosstalk is reflected not only in the cooperative or antagonistic interactions among distinct PTMs but also, more importantly, in their functional convergence within integrated resistance programs.

**Figure 1:**
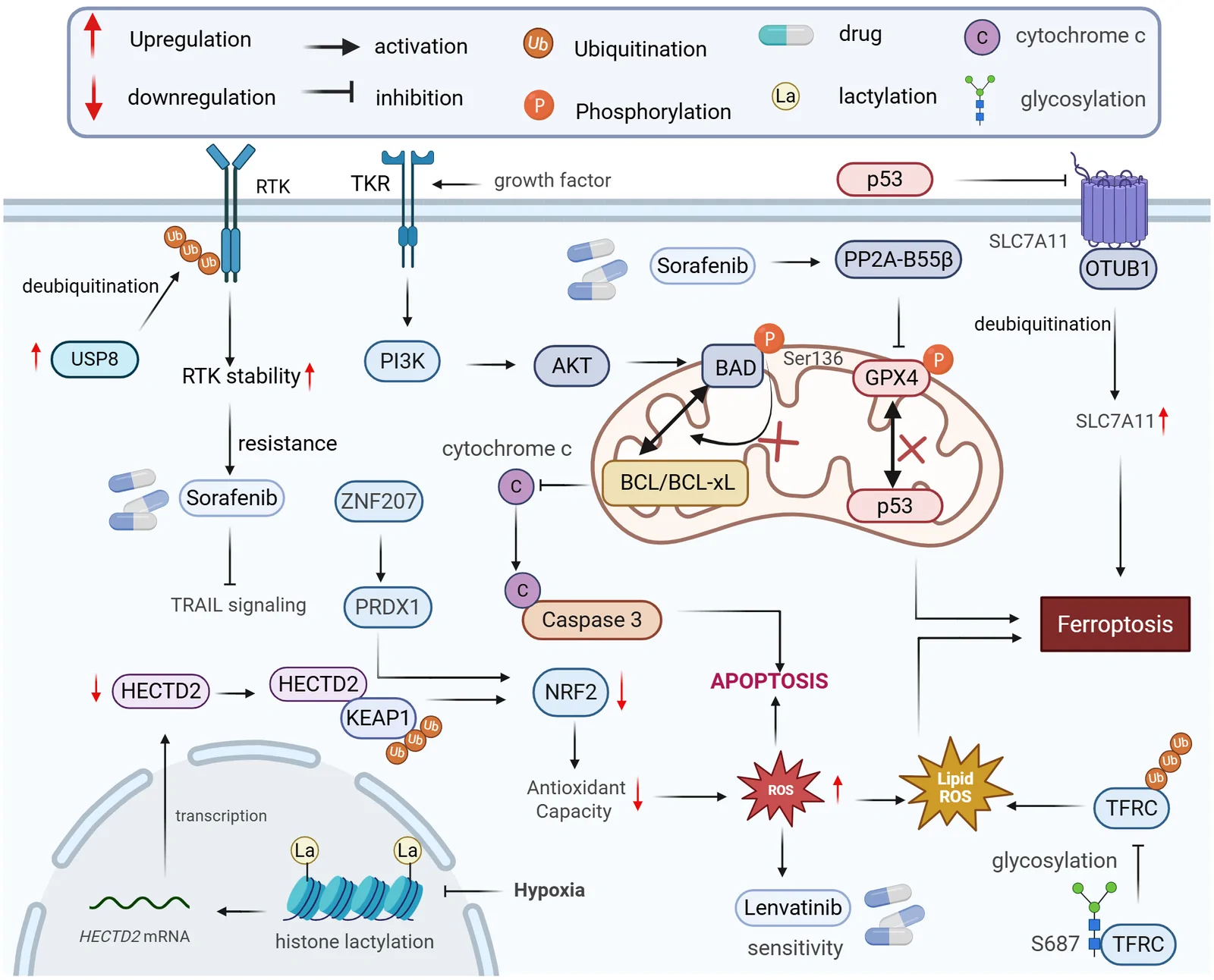
PTM-mediated multidimensional immunotherapy resistance network in hepatocellular carcinoma. This schematic diagram illustrates how post-translational modifications (PTMs) drive immune evasion across three major dimensions. Tumor cells: Antagonistic PTMs (e.g., glycosylation, ubiquitination, and phosphorylation) dynamically control the stability of immune checkpoints, such as PD-L1 and TIM-3. CD8^+^ T cells: Endogenous PTM networks drive terminal T cell exhaustion by impairing TCR signaling and inducing metabolic and lipid reprogramming. Tumor microenvironment (TME): Crosstalk between PTMs and metabolism (e.g., lactate-induced H3K18la) promotes the immunosuppressive functions of TAMs, Tregs, and MDSCs, while simultaneously impeding DC infiltration. Potential PTM-targeting inhibitors are also indicated. CAFs, Cancer-Associated Fibroblasts; MDSC, Myeloid-Derived Suppressor Cells; TAMs, Tumor-Associated Macrophages; TIM-3, T cell immunoglobulin and mucin domain-containing protein 3; ACVR2A, Activin A receptor type 2A; LTβR, Lymphotoxin beta receptor; TFRC, Transferrin Receptor; NUPR1, Nuclear protein 1; RBPJ, Recombination signal binding protein for immunoglobulin kappa J region; GMPS, Guanine monophosphate synthase; SENP3, Sentrin/SUMO-specific protease 3; MVP, Major vault protein; CMA, Chaperone-Mediated Autophagy.

Available evidence suggests that this integration converges mainly on two core dimensions: ferroptosis evasion and systemic stress adaptation. In terms of anti-oxidation, O-linked N-acetylglucosamine (O-GlcNAc) glycosylation upregulates solute carrier family 7 member 11 (SLC7A11) through the reactive oxygen species (ROS)–O-GlcNAc transferase (OGT)–forkhead box K2 (FOXK2) axis, directly inhibiting ferroptosis (43). Lactylation, on the other hand, utilizes the zinc finger protein 207 (ZNF207)-peroxiredoxin 1 (PRDX1)-nuclear factor erythroid 2-related factor 2 (NRF2) axis to further enhance the cell’s ability to clear active oxygen. Together, these mechanisms increase the tolerance of tumor cells to multikinase inhibitors (44). Notably, these seemingly independent signaling axes may interconnect through key nodes, such as the SLC7A11/xCT system and the NRF2 pathway, forming a dynamic drug resistance network that integrates metabolic reprogramming, oxidative stress response (45) (**Figure 1**).

Collectively, the drug resistance mechanism driven by PTM is a highly dynamic and interconnected system. Future studies should integrate multi-omics approaches to construct clinically relevant PTM interaction landscapes and elucidate the crosstalk among distinct PTMs. Building on these insights, the combination of highly selective PTM-targeting inhibitors with existing targeted therapies may provide opportunities to exploit synthetic lethal interactions and limit tumor adaptive evolution. Such strategies hold considerable promise for improving clinical outcomes in patients with advanced HCC (46).

## 4 PTMs and immunotherapy resistance in HCC

### 4.1 PTM-driven remodeling of tumor-intrinsic immune checkpoint signaling

The immune evasion capability of tumor cells is largely determined by the abundance and functional activity of cell-surface inhibitory receptors. Beyond the well-characterized PD-L1 pathway, PTM regulatory networks also govern the functional regulation of multiple immune checkpoint molecules, including LAG-3, TIGIT, TIM-3, and CTLA-4.The shared PTM regulatory mechanisms across these immune checkpoints collectively reinforce tumor immune evasion by sustaining multifaceted immunosuppressive states within the tumor microenvironment (47, 48).

Current studies indicate that the stability of PD-L1 is precisely regulated by two functionally opposing classes of PTMs. The first class comprises stabilizing modifications, mainly glycosylation and deubiquitination. N-Glycosylation mediated by the guanosine monophosphate synthetase (GMPS)/STT3 oligosaccharyltransferase complex catalytic subunit A (STT3A) pathway can shield the degradation recognition sites on PD-L1, allowing it to evade clearance by the ubiquitination-proteasome system (49). Ubiquitin-specific peptidase 47 (USP47) maintains PD-L1 stability by removing K48-linked ubiquitin chains (50). In addition, histone lactylation-mediated upregulation of major vault protein (MVP) can indirectly counteracts β-transducin repeat-containing protein (β-TrCP)-mediated degradation, while Sentrin/SUMO-specific protease 3 (SENP3) directly stabilizes PD-L1 homeostasis through deSUMOylation (51, 52). In contrast, the other type promotes PD-L1 degradation and limits its abnormal accumulation. Cyclin-dependent kinase 5 (CDK5)-mediated phosphorylation of PD-L1 at Thr290 triggers its association with heat shock cognate protein 70 (HSC70), leading to degradation via the chaperone-mediated autophagy (CMA) pathway (53). However, in immunotherapy-resistant tumors, this regulatory balance is often disrupted, with impaired degradation pathways and enhanced stabilization mechanismsjointly establishing the molecular basis of resistance.Furthermore, PTM-mediated regulation of PD-L1 may also be shaped by the etiological context of HCC. In HBV-associated HCC, HBx induces PD-L1 expression and promotes the establishment of an immunosuppressive tumor microenvironment, whereas AMPK activation facilitates RBX1-mediated ubiquitination and degradation of PD-L1, thereby enhancing CD8^+^ T-cell infiltration and increasing sensitivity to anti-PD-L1 therapy (54).

In HCC, the protein stability of TIM-3 is primarily maintained by palmitoylation at the Cys296 residue mediated by the palmitoyltransferase DHHC9. This modification competitively prevents the recruitment of the E3 ubiquitin ligase HRD1, thereby allowing TIM-3 to escape polyubiquitination-mediated degradation. Clinical analyses have demonstrated that DHHC9 expression in HCC tissues is positively correlated with TIM-3 levels on the surface of CD8^+^ T cells and NK cells, and that patients with high DHHC9 expression exhibit significantly poorer outcomes. These findings suggest that targeting TIM-3 palmitoylation may represent a potential strategy to restore antitumor immunity (55).

TIGIT is markedly upregulated in tumor-infiltrating lymphocytes in HCC and serves as an important marker of T-cell exhaustion. Pan-cancer analyses have demonstrated that N-glycosylation at the Asn101 residue determines the binding stability between TIGIT and its ligand PVR (56). In addition, LAG-3 and CTLA-4 are also extensively regulated by PTM networks. Ubiquitination of LAG-3 is essential for its inhibitory signaling function (57). N-glycosylation of CTLA-4 modulates its binding affinity toward the ligands CD80 and CD86, whereas its protein abundance is governed by ubiquitination-dependent lysosomal degradation pathways (58). Although the HCC-specific modification sites and regulatory mechanisms of these immune checkpoint molecules require further functional validation, accumulating evidence indicates that PTMs serve as fundamental regulatory mechanisms controlling their immunosuppressive functions.

Collectively, these immune checkpoint molecules exhibit shared regulatory patterns at the PTM level. Distinct types of modifications coordinately control protein stability and ligand-binding affinity, while PTMs at specific sites can antagonize ubiquitination-mediated degradation through steric hindrance. Targeting these regulatory nodes has emerged as a promising strategy to enhance immunotherapy responses in HCC. Representative approaches include disrupting PD-L1 glycosylation-mediated protection using Angustmycin A and promoting TIM-3 degradation through inhibition of DHHC9. Such precision interventions based on the regulation of protein homeostasis represent an emerging avenue for improving the efficacy of immunotherapy in HCC (50, 53, 59).

### 4.2 PTM-mediated regulation of T-cell exhaustion

In addition to tumor cell-mediated immune evasion, exhaustion of effector T cells represents another critical determinant of ineffective antitumor immunity (59). This exhausted state is not merely characterized by functional impairment but rather reflects a PTM-driven epigenetic reprogramming process, sustained by multiple biochemical cues within the HCC immune microenvironment.

One major mechanism involves suppression of T-cell receptor (TCR) signaling. In exhausted T cells from HCC, Src homology 2 domain-containing protein tyrosine phosphatase 1 (SHP-1) is markedly upregulated and suppresses T-cell activation by dephosphorylating key components of the TCR signaling cascade. Consistently, SHP-1-deficient T cells exhibit enhanced IFN-γ secretion and cytotoxic activity in humanized HCC patient-derived xenograft (PDX) models (60). A second mechanism involves metabolic elevation of the activation threshold. N-glycosylation of cluster of differentiation 36 (CD36) sustains recombination signal-binding protein for immunoglobulin kappa J region (RBPJ)-driven T-cell exhaustion by regulating AMPK phosphorylation and UFMylation of ubiquitin-specific peptidase 7 (USP7).Lipid metabolic reprogramming further promotes terminal transcriptional exhaustion (61). Riplet, also known as ring finger protein 135 (RNF135), is silenced by promoter hypermethylation; although this event is not a protein PTM, RNF135 loss impairs K48-linked polyubiquitination of fatty acid synthase (FASN), thereby accelerating fatty acid production. Free fatty acids enhance STAT3 palmitoylation in T cells, leading to activation of signal transducer and activator of transcription 3 (STAT3) and ultimately driving terminal exhaustion of CD8+ T cells (62).

Collectively, PTMs reinforce T-cell exhaustion through coordinated regulation of signaling initiation, metabolic sensing, and transcriptional programming. This regulatory paradigm extends beyond T cells to other effector immune populations, including NK cells. In HCC-infiltrating NK cells, the TIGIT/CD155 axis suppresses STAT3/GLUT1-associated glycolytic metabolism in an SHP-2-dependent manner, resulting in impaired NK-cell function. Targeting this PTM-dependent regulatory axis not only restores NK-cell activity but also, together with T-cell-directed strategies, provides an integrated approach to reinvigorating the antitumor potential of effector lymphocytes (63).Taken together, effective restoring effector immune function is unlikely to be achieved through PD-1 blockade alone; concurrent targeting of PTM-dependent suppressive programs may represent an important complementary strategy.

### 4.3 PTM-mediated immune resistance in the tumor microenvironment

The development of immune resistance extends beyond the direct crosstalk between tumor cells and T cells and involves extensive remodeling of the tumor microenvironment (TME) (64, 65). Within this context, PTMs serve as critical regulatory mechanisms that coordinate multiple cellular and molecular processes across the TME.

Tumor-derived lactate generated by hyperactive metabolism induces histone H3 lysine 18 lactylation (H3K18la) in tumor-associated macrophages (TAMs), leading to upregulation of nuclear protein 1 (NUPR1) and subsequent activation of the ERK/JNK signaling pathway, thereby promoting M2 polarization of TAMs and establishing a self-reinforcing immunosuppressive circuit. Pharmacological targeting of NUPR1 has been shown to reverse M2 polarization and enhance the efficacy of anti-PD-1 therapy (66).

The recruitment and activation of myeloid-derived suppressor cells (MDSCs) are tightly governed by post-translational regulatory mechanisms.Activation of the JAK–STAT3 signaling pathway is a key regulatory mechanism driving MDSC expansion and the acquisition of immunosuppressive functions (67). In HCC, cancer-associated fibroblasts facilitate MDSC generation and activation through IL-6 secretion, thereby triggering STAT3 phosphorylation and downstream immunosuppressive programs (68). Meanwhile, activation of the IL-8/CXCR2 signaling axis drives MDSC trafficking and immunosuppressive activity, and targeting this pathway enhances sensitivity to anti-PD-L1 therapy (69). Furthermore, CCRK activates the STAT3/E4BP4 signaling axis in MDSCs, inducing IL-10 expression and suppressing IL-12 production at the transcriptional level, thereby conferring immunosuppressive properties on MDSCs (70). Collectively, these PTM-associated regulatory events establish a suppressive defense network mediated by MDSCs within the tumor microenvironment.

Notably, activin A receptor type 2A (ACVR2A) inactivation-mediated glycolytic reprogramming recruits Foxp-3^+^ regulatory T cells (Treg), representing another critical mechanism underlying tumor immune evasion and resistance to PD-1 blockade (71). In parallel, the stability of FOXP3 is dually regulated by by retinoblastoma-binding protein 6 (RBBP6)-mediated ubiquitination and lymphotoxin-β receptor (LTβR)-dependent glycosylation, thereby fine-tuning the suppressive activity of Tregs (72, 73).

**Figure 2:**
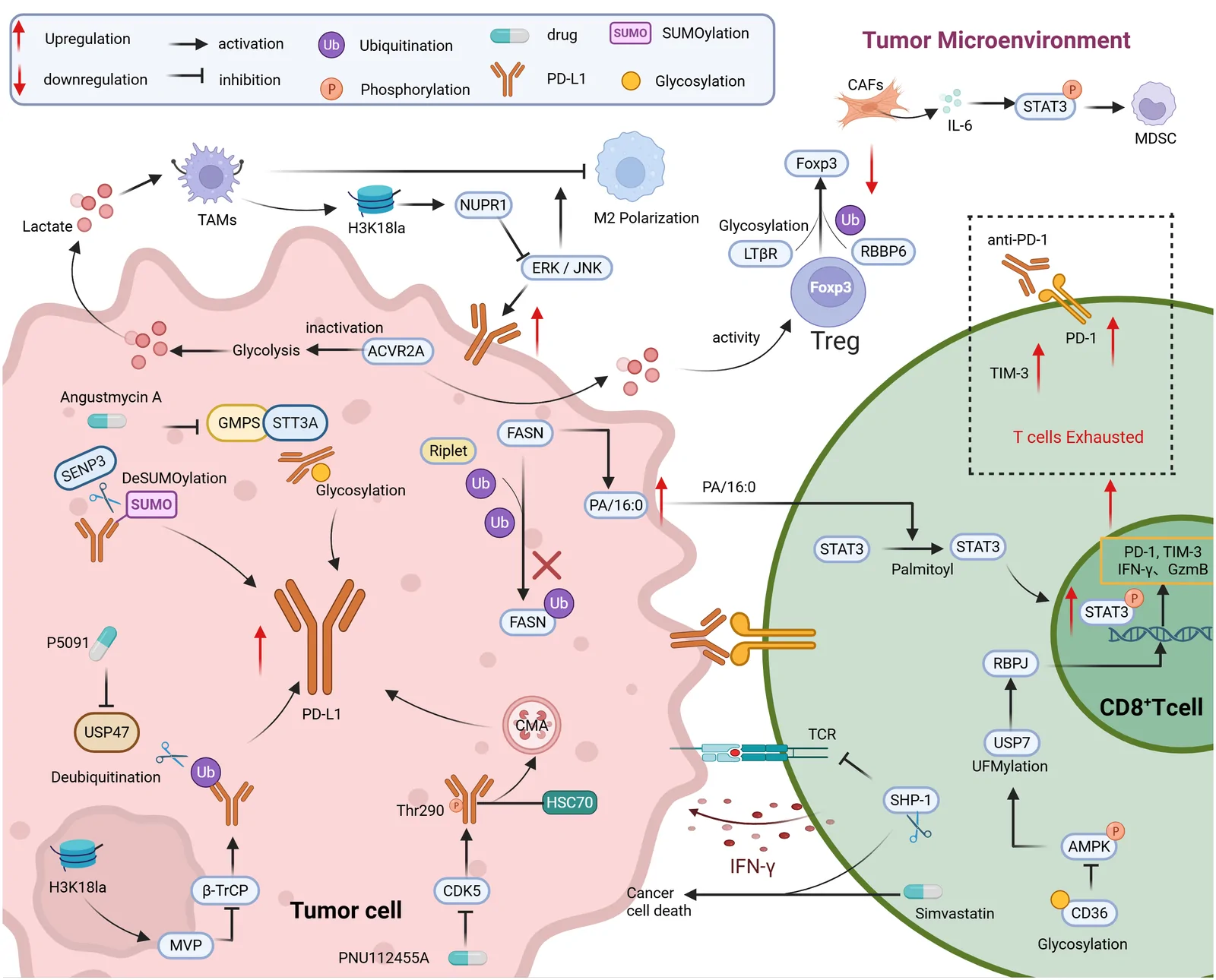
PTMs drive HCC resistance by regulating programmed cell death. Ubiquitin-Specific Protease 8 (USP8)-mediated Receptor Tyrosine Kinase (RTK) deubiquitination and Protein Kinase B (AKT)-driven Bcl-2-associated death promoter (BAD) phosphorylation promote survival and sorafenib resistance, while ferroptosis is co-regulated by OTU domain-containing ubiquitin aldehyde-binding protein 1 (OTUB1), Protein Phosphatase 2A-B55β (PP2A-B55β), and Transferrin Receptor (TFRC) glycosylation, with these PTM networks collectively modulating HCC drug resistance.

Dysfunction of dendritic cells (DCs) represents another critical component of tumor immune evasion. Aberrant activation of the Wnt/β-catenin pathway suppresses CCL5 expression, thereby limiting DC infiltration into tumor tissues and contributing to T-cell exclusion (74). Although β-catenin itself is precisely regulated by phosphorylation and ubiquitination, whether PTMs directly govern the antigen-presenting capacity of DCs remains largely unexplored. Collectively, PTMs orchestrate multicellular regulatory networks that promote the establishment of an immunosuppressive (“cold”) tumor microenvironment (**Figure 2**).

### 4.4 PTM-mediated crosstalk and adaptive cross-resistance during combination therapy

The therapeutic efficacy of combining targeted agents with ICIs is not simply a result of additive effects between two independent mechanisms. The recently proposed “metabolic bridge” framework suggests that targeted therapy-induced stress and efferocytosis cooperate to remodel the metabolic landscape of the HCC tumor microenvironment. TKI-induced tumor cell death generates abundant apoptotic substrates; when these substrates exceed the mitochondrial processing capacity of macrophages, metabolic flux is redirected toward lactate accumulation. Lactate then functions as a central signaling hub, driving tumor-intrinsic resistance and microenvironmental immunosuppression through diverse lactylation-mediated regulatory events (75).

At the level of targeted therapy resistance, lactate derived from GPX8^+^ cancer-associated fibroblasts is transported into HCC cells through MCT1, where it induces histone H3K18 lactylation (H3K18la)-mediated transcriptional upregulation of BRPF1, subsequently activating EGFR signaling and conferring resistance to lenvatinib (76). Meanwhile, bidirectional regulation of H4K12 lactylation by AARS1 and HDAC11 directly activates the RAPGEF3–RAP1 signaling axis, thereby driving acquired resistance to targeted therapies (77). More profound crosstalk emerges through metabolic feedback between distinct PTM modalities. JOSD1-mediated deubiquitination and AARS1-mediated lactylation are directly coupled to cooperatively maintain the stability and activity of the glycolytic enzyme PGAM1, forming a self-reinforcing “metabolism–PTM–metabolism” circuit that further promotes lactate accumulation and microenvironmental immunosuppression (78).

PTM-mediated metabolic remodeling also influences responses to immunotherapy. Lactate-induced ATF6 lactylation at K424 (K424la) prevents its ubiquitination-mediated degradation, thereby activating the TDO2–kynurenine pathway and enhancing regulatory T-cell (Treg) function. Conversely, Treg-derived extracellular NAMPT (eNAMPT) promotes glycolytic acid production in tumor cells, establishing a closed-loop “metabolism–PTM–immune–metabolism” circuit that reinforces immunosuppression (79). As described above, lactate also induces H3K18 lactylation to upregulate NUPR1 expression in tumor-associated macrophages (TAMs), ultimately promoting CD8^+^ T-cell exhaustion and resistance to immunotherapy (66).

Notably, the selective pressure imposed by immunotherapy can reciprocally reshape tumor sensitivity to targeted agents. Persistent interferon signaling has been shown to drive epigenetic reprogramming of tumor cells through a STAT1 phosphorylation-dependent mechanism, leading to the upregulation of multiple immunosuppressive ligand molecules and the establishment of dual resistance barriers involving both PD-L1-dependent and PD-L1-independent mechanisms (80). This signaling adaptation also provides tumor cells with additional PTM-dependent survival cues, thereby promoting cross-resistance to TKIs (81).

This complex molecular crosstalk suggests that future combination strategies should identify and simultaneously target key nodes driving cross-resistance within the PTM regulatory network. Integrating PD-L1-degrading agents, such as the GMPS inhibitor Angustmycin A, with agents that reverse T-cell exhaustion, such as the SHP2 inhibitor JAB-3312, and lactylation-targeting interventions that remodel the tumor metabolic microenvironment may represent a promising therapeutic approach (49, 82). Such combination strategies aim to weaken tumor-mediated immune defense while enhancing immune-mediated antitumor activity, providing a feasible clinical avenue for overcoming immunotherapy resistance in HCC.

## 5 Post-translational modifications in resistance to locoregional therapies for HCC

### 5.1 Roles of PTMs in SBRT resistance

Stereotactic body radiotherapy (SBRT) exerts its antitumor effects by inducing DNA double-strand breaks (DSBs) through high-dose ionizing radiation. However, HCC cells often exploit PTM-regulated repair mechanisms to establish efficient radioprotective responses.

During non-homologous end joining (NHEJ) repair, TKT interacts with PARP1 and undergoes PARylation-mediated enzymatic inhibition, redirecting metabolic flux toward DNA repair. Meanwhile, TKT functions as a non-enzymatic scaffold to enhance PARP1 auto-PARylation, thereby cooperatively promoting the efficiency of both homologous recombination (HR) and NHEJ repair pathways (83). PC4 further facilitates NHEJ repair through PARylation-dependent enhancement of the Ku complex (84). In addition, radiation-induced O-GlcNAcylation of NONO promotes its regulation of SETMAR alternative splicing, generating a long isoform that subsequently recruits Ku70 to initiate NHEJ repair (85).

At the level of homologous recombination (HR) repair, succinylation regulates HR efficiency by modulating splicing events. KAT2A-mediated succinylation of SRSF11 stabilizes the interaction between SRSF11 and the spliceosome, promoting retention of exon 10 in RAD52 and preserving its RAD51-binding domain, thereby enhancing HR repair and conferring radioresistance (86). In addition, UBAP2 promotes ubiquitination-mediated degradation of SLC27A5, resulting in increased RAD51 expression and selective enhancement of the HR pathway (87). Lactylation of MRE11 enhances its DNA-binding capacity and end-resection activity, thereby augmenting HR repair efficiency; this mechanism has been demonstrated across multiple cancer types to promote resistance to radiotherapy and chemotherapy (88). Collectively, these PTM events cooperate to enable HCC cells to rapidly repair sublethal radiation-induced DNA damage.

### 5.2 Roles of PTMs in TACE resistance

Transarterial chemoembolization (TACE) induces ischemic necrosis through arterial embolization and local chemotherapy delivery. However, the hypoxic microenvironment and chemotherapeutic stress following embolization drive residual HCC cells to activate adaptive survival programs, in which PTMs enhance the survival capacity of residual tumor cells.

In TACE-associated resistance, the TKT/c-Myc positive feedback loop is activated. TKT promotes c-Raf phosphorylation at Ser338 and subsequent ERK activation, thereby enhancing c-Myc phosphorylation at Ser62. This phosphorylation event stabilizes c-Myc protein and drives rapid postoperative tumor regrowth (89). Meanwhile, SERPINA1 competitively binds to ITGB3 and blocks the ITCH-mediated ubiquitination and degradation pathway, thereby sustaining pro-survival signaling (90). Furthermore, oxidative stress-induced activation of PKCδ phosphorylates G6PD at Thr236, which subsequently facilitates SUMOylation at Lys238. This modification enhances pentose phosphate pathway flux, maintains redox homeostasis, and protects residual HCC cells from oxidative stress-induced damage following TACE (91).

### 5.3 PTM-mediated remodeling of the immune microenvironment during locoregional therapy

Locoregional therapies can induce immunogenic cell death (ICD), leading to the release of tumor antigens and theoretically eliciting antitumor immune responses. For example, combination of the histone deacetylase (HDAC) inhibitor vorinostat with radiotherapy increases the expression of NKG2D ligands on the surface of HCC cells, thereby enhancing immune recognition by NK cells (92).

However, PTM-mediated remodeling of the tumor microenvironment often converts this potential for immune activation into an immunosuppressive state. For example, MORF4L1 acetylates PALB2 at lysine 628 (K628), thereby preventing its ubiquitination and degradation while simultaneously enhancing histone H3K4 acetylation to promote DNA repair. Pharmacological inhibition of MORF4L1 with argatroban in combination with radiotherapy induces cytosolic DNA accumulation by impairing DNA repair, thereby activating the cGAS–STING pathway and enhancing antitumor immune responses (93). Furthermore, histone lactylation in the TACE-induced microenvironment promotes polarization toward tumor-promoting SPP1^+^ macrophages, generating a potent immunosuppressive niche that counteracts the benefits of tumor antigen release following TACE (94).

Once this PTM-driven dynamic equilibrium shifts toward immune evasion, the abscopal effects of SBRT and the immunological benefits derived from TACE-induced antigen release become difficult to fully achieve, thereby limiting the synergy between locoregional therapy and systemic immunotherapy (**Table 1**).

**Table 1:** PTMs in HCC Drug Resistance.

Drug	Target	PTM type	Modifying enzyme/regulatory factor	Mechanism	Intervention strategy	Reference
Oxaliplatin	LOXL3 (S704)	Phosphorylation	AK2	AK2/LOXL3/DHODH/ferroptosis/resistance	Leflunomide	(15)
	IGF2BP1 (R452)	Methylation	PRMT3	PRMT3/IGF2BP1/HEG1/survival signaling/resistance	PRMT3 as biomarker	(16)
5-FU	SMC4	Deubiquitination	USP39	USP39/SMC4/cell cycle/proliferation/resistance	SMC4/USP39 knockdown	(19)
	YIPF5	Ubiquitination	(Mitophagy)	YIPF5/mitophagy/senescence/5-FU sensitivity	Salidroside	(20)
FOLFOX	HELLS	Deubiquitination & SUMOylation	USP1	USP1/HELLS/EMT/HRR/chemoresistance	ML323	(17)
	Histone H3 (K14)	Lactylation	p300 (EP300)	p300/H3K14la/NEDD4/PTEN/AKT/resistance	Glycolysis inhibitors	(21)
	IGF2BP3 (K76)	Lactylation	PARK7	PARK7/IGF2BP3/FSP1/ferroptosis/resistance	Blocking antibody	(22)
Sorafenib	PCK1 (K473)/PCK2 (K491)	Acetylation	KAT8	KAT8/PCK1/PCK2/metabolic transition/resistance	PCK2 inhibition	(23)
	GLS (Y308/S314)	Phosphorylation & Ubiquitination	S100A14	S100A14/GLS/oxidative stress/apoptosis/resistance	GLS inhibition	(24)
	SIRT1/AMPK	Deacetylation & Phosphorylation	SIRT1	NAD/SIRT1/AMPK/mTOR/apoptosis/resistance	SIRT1 inhibition	(25)
	NPM1 (K54)	Acetylation	XPO1	XPO1/NPM1/Vimentin/EMT/resistance	KPT-8602	(26)
	PLEKHG5	Deacetylation	HDAC2	HDAC2/PLEKHG5/Rac1/AKT/NF-kB/resistance	HDAC2 inhibitors	(27)
	ITGB5/EGFR	Phosphorylation (ITGB5)	CSNK1A1/EPS15	ITGB5/EPS15/EGFR/CSNK1A1/AKT/mTOR/resistance	Targeting ITGB5/CSNK1A1 axis	(28)
	CHK2/BRCA1	Phosphorylation	NOTCH1/ATM axis	NOTCH1/ATM/CHK2/BRCA1/genomic stability/resistance	ATM inhibition	(29)
	CDK11B/H2B (K120)	Deubiquitination	USP22	USP22/CDK11B/H2B/TFRC/ferroptosis/resistance	Hyperoside	(30)
	NCOA4	DeISGylation	USP18	USP18/NCOA4/ferroptosis/acquired resistance	Hyperoside	(31)
	CCT3 (K21)	K6-linked Deubiquitination	USP11	USP11/CCT3/ACTN4/TFRC/ferroptosis/resistance	CCT3/USP11 inhibition	(32)
	GPX4 (S2)	Dephosphorylation	PP2A-B55β	PP2A-B55β/GPX4/ferroptosis/resistance	LB-100 (PP2A inhibitor)	(33)
Lenvatinib	EGFR	N-glycosylation	DDOST	DDOST/EGFR/survival signaling/acquired resistance	DDOST inhibition	(38)
	CRTC2	Dephosphorylation	PP2A	PP2A/CRTC2/CREB/survival genes/resistance	CRTC2 inhibition	(39)
	LGALS3	Deubiquitination	USP15	USP15/LGALS3/AKT/mTOR/resistance	LGALS3/USP15 inhibition	(40)
	HSD17B4	Ubiquitination (Degradative)	DTX2	DTX2/HSD17B4/lipid metabolism/ferroptosis/resistance	DTX2 inhibition	(41)
Regorafenib	RPL14 (R141)	Symmetric dimethylation	PRMT5	PRMT5/RPL14/DNA damage/resistance	PRMT5 inhibition	(42)
Multikinase Inhibitors	FOXK2/SLC7A11	O-GlcNAcylation	OGT	ROS/OGT/FOXK2/SLC7A11/ferroptosis/resistance	OGT inhibitors	(43)
	PRDX1 (K67)/NRF2	Lactylation	ZNF207	ZNF207/PRDX1/NRF2/antioxidant/ferroptosis/resistance	NRF2 inhibition	(44)
Immunotherapy	PD-L1	N-glycosylation	STT3A/GMPS	GMPS/STT3A/PD-L1/CD8+ T cell/immune evasion	Angustmycin A	(49)
	PD-L1	Deubiquitination	USP47	USP47/PD-L1/protein stability/immune evasion	P5091	(50)
	PD-L1/MVP	Lactylation & Ubiquitination	MVP/beta-TrCP	Lactylation/MVP/PD-L1/beta-TrCP/immune evasion	Lactylation inhibitors	(51)
	PD-L1 (T290)	Phosphorylation	CDK5/HSC70	CDK5/PD-L1/T290/HSC70/CMA/immune evasion	PNU112455A	(53)
	TIM-3 (C296)	Palmitoylation	DHHC9/HRD1	DHHC9/TIM-3/HRD1/immune evasion/resistance	DHHC9 knockdown	(55)
	SHP-1	Dephosphorylation	SHP-1	SHP-1/TCR signaling/HMGCR/T-cell exhaustion	SHP-1-KO T cells	(60)
	CD36/RBPJ	N-glycosylation/UFMylation	USP7/AMPK	CD36/AMPK/USP7/RBPJ/T-cell exhaustion/evasion	Targeting USP7/RBPJ	(61)
	FASN/STAT3	Palmitoylation	Riplet/RNF135	Riplet/FASN/palmitic acid/STAT3/T-cell exhaustion	FASN inhibitors	(62)
	STAT3 (Y705)	Dephosphorylation	SHP-2/TIGIT	TIGIT/SHP-2/STAT3/GLUT1/glycolysis/resistance	anti-TIGIT/SHP099/JSI-124	(63)
	H3K18la (TAMs)/NUPR1	Lactylation	Lactate (Tumor-derived)	Lactate/H3K18la/NUPR1/M2 polarization/evasion	Targeting NUPR1	(66)
	STAT3	Phosphorylation	IL-6/SDF-1α	TAFs/SDF-1α/CXCR4/IL-6/STAT3/MDSC induction/immune suppression	SDF-1α inhibition	(68)
	STAT3 (Y705)	Phosphorylation	CCRK	CCRK/STAT3/E4BP4/IL-10/IL-12/immune suppression	Stattic/Ccrk-knockdown	(70)
	SMAD2/3/Foxp3	Phosphorylation & Lactylation	ACVR2A/Lactate	ACVR2A/SMAD/MCT4/lactate/Foxp3/Treg/evasion	MCT4 inhibitors	(71)
	LTβR/RORC/Foxp3	N-glycosylation & Ubiquitination	PELI1/SMURF1	LTβR/PELI1/RORC/Foxp3/Th17/Treg/ratio/evasion	LTβR-CAR-T + 2-DG	(73)
Lenvatinib (TKI)	Histone H3 (K18la)	Lactylation	BRPF1	GPX8/lactate/MCT1/H3K18la/BRPF1/H3K14ac/EGFR/resistance	AZD3965; GSK5959	(76)
Sorafenib/Lenvatinib (TKIs)	Histone H4 (K12la)	Lactylation	AARS1/HDAC11	AARS1/H4K12la/RAPGEF3/RAP1/resistance	ESI-09	(77)
Anti-PD-1 (ICI)	PGAM1 (K251)	Ubiquitination & Lactylation	JOSD1/AARS1	JOSD1/AARS1/PGAM1/PTM crosstalk/glycolysis/immune suppression	JOSD1 inhibition + anti-PD-1 therapy	(78)
Anti-PD-1/PD-L1 (ICI)	ATF6 (K424la)	Lactylation	AARS1	AARS1/ATF6/K424la/TDO2/kynurenine/Treg/glycolysis/immune resistance	β-alanine+ PD-1/PD-L1 blockade	(79)
Radiotherapy (RT)	TKT	PARylation	PARP1	PARP1/TKT/HR-NHEJ/DNA repair/radioresistance	TKT depletion or PARP1 inhibition	(83)
	Ku complex	PARylation	PC4/PARP1	PC4/PARP1/Ku complex/NHEJ/DNA repair/radioresistance	PC4 targeting	(84)
	NONO (S147)	O-GlcNAcylation	NONO/SFPQ/SETMAR	NONO/SETMAR splicing/Ku70/NHEJ/DNA repair/radioresistance	Targeting NONO O-GlcNAcylation	(85)
	SRSF11 (K419)	Succinylation	KAT2A	KAT2A/SRSF11/RAD52 splicing/RAD51-HR/DNA repair/radioresistance	KAT2A-SRSF11 axis disruption	(86)
	SLC27A5	Ubiquitination	UBAP2	UBAP2/SLC27A5/RAD51/HR/DNA repair/radioresistance	Targeting UBAP2/SLC27A5 axis	(87)
TACE	c-Raf (S338)/c-Myc (S62)	Phosphorylation	TKT/RAF1	TKT/RAF1/ERK/c-Myc/stability/proliferation/TACE resistance	Targeting TKT/c-Myc axis	(89)
	ITGB3	Ubiquitination	SERPINA1/ITCH	SERPINA1/ITGB3/ITCH/proteasomal degradation/oncogenic signaling/TACE resistance	Targeting SERPINA1/ITGB3 axis	(90)
	G6PD (T236/K238)	Phosphorylation & SUMOylation	PKCδ/SENP1	PKCδ/G6PD/PPP/redox balance/survival/TACE resistance	PKCδ inhibition	(91)
Radiotherapy (immune modulation)	PALB2 (K628)/Histone H3 (K4)	Acetylation	MORF4L1	MORF4L1/PALB2/H3K4/DNA repair/immune response/radioresistance	Argatroban + RT	(93)
TACE (immune modulation)	Macrophage/SPP1+ macrophages	Lactylation	Lactate metabolism	Lactate/SPP1+ macrophage/CD8+ T cell dysfunction/immune suppression/TACE resistance	Targeting lactate-induced lactylation or SPP1-CD44 axis	(94)

## 6 PTM-driven tumor persistence and distant recurrence in HCC

### 6.1 PTM-mediated regulation of tumor subclonal evolution

Under prolonged therapeutic pressure, PTMs drive functional rewiring of metabolic enzymes and directly reshape the chromatin landscape, establishing drug-resistant epigenetic imprints in specific tumor subclones. This process enables these subclones to acquire persistent, mutation-independent competitive advantages without requiring alterations in the underlying genetic material.

A representative example involves the glycolytic enzyme pyruvate kinase M2 (PKM2). EGFR–ERK-mediated phosphorylation of PKM2 at Ser37 not only suppresses its enzymatic activity and sustains the Warburg phenotype but also promotes its nuclear translocation and subsequent phosphorylation of histone H3 at Thr11, thereby directly linking metabolic state to transcriptional regulation (95, 96). This mechanism bypasses conventional transcription factor-dependent pathways, enabling subclones to rapidly reprogram chromatin accessibility under TKI pressure. Meanwhile, ATP citrate lyase (ACLY), a central enzyme in lipid biosynthesis, is activated through AKT-mediated phosphorylation and serves as a major source of cytosolic acetyl-CoA (97). ACLY is further regulated through multiple PTMs. Chemotherapy-induced N-acetyltransferase 10 (NAT10)-mediated acetylation at Lys468 enhances its activity, whereas the hypoxic microenvironment stabilizes ACLY through HIF-1α–USP13-dependent deubiquitination (98, 99). These regulatory events not only support the increased membrane biosynthetic demands of resistant cells but also provide a critical source of acetyl groups for epigenetic remodeling. Evidence from colorectal cancer has shown that phosphorylation-mediated activation of ATP citrate lyase (ACLY) increases nuclear acetyl-CoA availability, which subsequently serves as a substrate for acetyltransferases such as p300 to catalyze histone H3 lysine 27 acetylation (H3K27ac) at the promoters of stemness-associated genes, including NANOG, thereby promoting their transcriptional activation (100). It should be noted that this regulatory axis is currently supported mainly by evidence from colorectal cancer models, and its direct validation in HCC remains limited. Nevertheless, it provides important clues for understanding the coupling between metabolic adaptation and stemness maintenance in HCC (101).

Notably, beyond epigenetic reprogramming, long-term survival of tumor subclones under therapeutic pressure also requires PTM-mediated remodeling of survival thresholds. NRF2 and p53 function as universal regulatory hubs integrating multiple cell death pathways. NRF2 serves as a guardian of redox homeostasis, and its stability is tightly controlled by KEAP1-mediated ubiquitination. Under oxidative stress, KEAP1 inactivation leads to NRF2 accumulation and nuclear translocation, inducing the expression of antioxidant enzymes such as SLC7A11 and thereby increasing the tolerance threshold toward both apoptosis and ferroptosis (102–104). In contrast, the functional outcome of p53 is determined by its site-specific PTM status (105). DNA damage-induced C-terminal acetylation, such as K382 acetylation, preferentially activates the transcription of pro-apoptotic genes including PUMA and Bax (106), whereas the zinc finger protein ZNF498 directly interacts with p53 and suppresses its Ser46 phosphorylation, thereby attenuating p53-mediated apoptosis and ferroptosis (107). This PTM-dependent functional switching of p53 enables resistant subclones to evade therapy-induced cell death. The flexible regulation of p53, together with the broad protective function of NRF2, allows resistant subclones to adaptively adjust survival strategies under therapeutic stress, forming a core PTM-mediated basis for tumor persistence (101).

Through integrating metabolic signals, chromatin modifications, and survival regulatory hubs into a coordinated network, PTMs enable HCC subclones under therapeutic pressure to acquire heritable phenotypic plasticity without altering their genomic sequence, providing a fundamental molecular basis for long-term tumor persistence.

### 6.2 PTMs in CSC maintenance

Under sustained therapeutic pressure, residual tumor cells undergoing EMT acquire metastatic potential, and the survival and colonization capacity of circulating tumor cells (CTCs) become critically dependent on PTM-mediated regulation of stemness-associated transcriptional programs.

The stability of c-Myc is precisely controlled by a phosphorylation-dependent balance. ERK-mediated phosphorylation at Ser62 stabilizes c-Myc, whereas glycogen synthase kinase 3β (GSK-3β)-mediated phosphorylation at Thr58 promotes its degradation (108, 109). This dynamic balance enables circulating tumor cells (CTCs) to rapidly respond to survival signals within the circulating microenvironment and transition between proliferative and survival states. In addition, ubiquitin-specific peptidase 22 (USP22) sustains c-Myc stability by preventing its proteasomal degradation, thereby promoting CSC self-renewal programs (110).

The nuclear accumulation of the Wnt signaling effector β-catenin is tightly regulated by the phosphorylation status of its N-terminal domain. Disruption of PTM-dependent degradation mechanisms impairs β-catenin ubiquitination and turnover, thereby facilitating transcription of downstream stemness-associated genes (111). This phosphorylation-dependent nucleocytoplasmic regulation sustains CTC stemness during circulation and primes metastatic colonization at distant sites.Etiological factors further reshape β-catenin-associated ubiquitination networks. In HBV-associated HCC, HBx induces MYH9 expression and promotes GSK3β ubiquitination-mediated degradation, disrupting the β-catenin destruction complex and enhancing stemness, EMT, and sorafenib resistance (112). This mechanism highlights the role of HBV-driven PTM remodeling in CSC maintenance and therapeutic adaptation. Moreover, reduced phosphorylation of the Hippo effectors YAP/TAZ promotes their nuclear localization and transcriptional cooperation with β-catenin, establishing a regulatory axis governing CTC stemness and metastatic plasticity (113).

These PTM events function as an integrated regulatory network. Through coordinated control of phosphorylation dynamics, deubiquitination-mediated stabilization, and nucleocytoplasmic trafficking, PTMs sustain stemness transcriptional programs, enabling CTC survival in circulation and distant organ colonization as a basis for tumor recurrence.

### 6.3 PTM-driven tumor cell dormancy and late recurrence

Late distant metastasis occurring years or even decades after treatment suggests that disseminated tumor cells (DTCs) can enter a reversible G0 dormant state to evade immune surveillance and therapeutic elimination. PTMs act as key molecular switches governing tumor dormancy.

Phosphorylation signaling represents a central regulatory axis governing the dormancy–reactivation switch. The ERK/p38 MAPK activity ratio determines cell fate, with high p38/ERK activity favoring dormancy. In HCC, restoration of miR-122 expression induces stem-like cancer cells to enter a dormant state, accompanied by p38 MAPK activation and a decreased p-ERK/p-p38 ratio (114). At the molecular execution level, CAV1-mediated phosphorylation networks provide a mechanistic basis for long-term DTC dormancy in HCC. CAV1 inhibition downregulates dormancy-associated regulators, including E-cadherin, RAC1, and p21, disrupts downstream STAT3/P70S6K and AMPKα signaling, and drives dormant cancer cells toward reactivation followed by autophagic cell death. Single-cell sequencing has identified a subset of CAV1^+^ recurrence-associated cancer cells in HCC patients, suggesting that PTM-maintained phosphorylation homeostasis is a key determinant of long-term DTC persistence (115).

Deubiquitination further regulates the balance between dormancy and senescence. WDR20 acts as an activator of the deubiquitinases USP12/46, promoting c-Myc deubiquitination and stabilization to prevent HCC cells from entering irreversible senescence and preserve the reactivation potential of DTCs (116). Collectively, HCC DTC dormancy represents an actively regulated adaptive state, with phosphorylation, ubiquitination, and other PTM events orchestrating the molecular programs underlying late recurrence (**Table 2**).

**Table 2:** PTM-mediated molecular events driving HCC persistence and recurrence.

PTM type	Target molecule	Upstream	Mechanism	Biological outcome	Reference
Phosphorylation	PKM2 (S37)/Histone H3 (T11)	EGFR -ERK	EGFR/PKM2/H3-T11/chromatin remodeling	Metabolic-epigenetic adaptation and subclonal persistence under therapeutic stress	(96)
	p53 (S46)	ZNF498	ZNF498/p53/S46 phosphorylation/apoptosis/ferroptosis	Survival advantage; tumor persistence	(107)
	c-Myc (S62)	TKT	TKT/RAF1/ERK/c-Myc	c-Myc stability; stemness maintenance	(108)
	c-Myc (S62/T58)	TTC36	TTC36/PP2A/GSK3b/c-Myc	c-Myc stabilization; CSC maintenance and survival advantage	(109)
	β-catenin (S33/S37/T41/S45)	GSK-3β	GSK-3β/β-catenin phosphorylation/Wnt signaling	β-catenin stabilization; HCC progression	(109)
	YAP/TAZ	Hippo pathway (MST1/2)	Hippo/YAP/TAZ/Wnt signaling	YAP/TAZ activation; tumor cell plasticity	(113)
	ERK/p38 MAPK	miR-122	miR-122/p38 MAPK/ERK phosphorylation balance	Low p-ERK/p-p38 ratio induces tumor dormancy program	(114)
	STAT3/P70S6K/AMPKα	CAV1	CAV1/STAT3/P70S6K/AMPKα phosphorylation signaling	Dormancy maintenance; recurrence-associated cell survival	(115)
Ubiquitination	KEAP1/NRF2	HECTD2	HECTD2/KEAP1/NRF2/redox adaptation/resistance	Oxidative stress tolerance; resistant subclone survival	(102)
Deubiquitination	ACLY(K726)	HIF-1α/USP13	HIF-1a/USP13/ACLY/stability/metabolic adaptation	Hypoxia adaptation and survival advantageDrives ferroptosis resistance and tumor immune evasion	(98)
	FKBP12	USP22	USP22/FKBP12/mTORC1/autophagy	Promotes HCC tumorigenesis and stemness	(110)
	c-Myc	WDR20/USP12/46	WDR20/USP12/46/c-Myc deubiquitination/stability	Senescence avoidance; HCC cell survival maintenance	(116)
Acetylation	ACLY (K468)	NAT10	NAT10/ACLY/H3K27ac/nuclear acetyl-CoA	Metabolic-epigenetic remodeling and chemoresistant clone persistence	(99)
	p53 (K382)	CBP/p300	p53/K382 acetylation/apoptotic transcription	Apoptosis induction; stress response regulation	(1)
Histone Lactylation	HECTD2 (Promoter)	Lactate	Lactate/H3K18la/HECTD2/NRF2	Upregulates HECTD2; drives lenvatinib resistance	(102)

## 7 Clinical translation and therapeutic strategies targeting PTM networks

The dynamic balance of PTMs is governed by a coordinated network of “writers,” “erasers,” and “readers.” The reversible nature of these modifications provides a unique opportunity for therapeutic intervention and has emerged as a promising foundation for overcoming therapeutic resistance in HCC (117).

**Figure 3:**
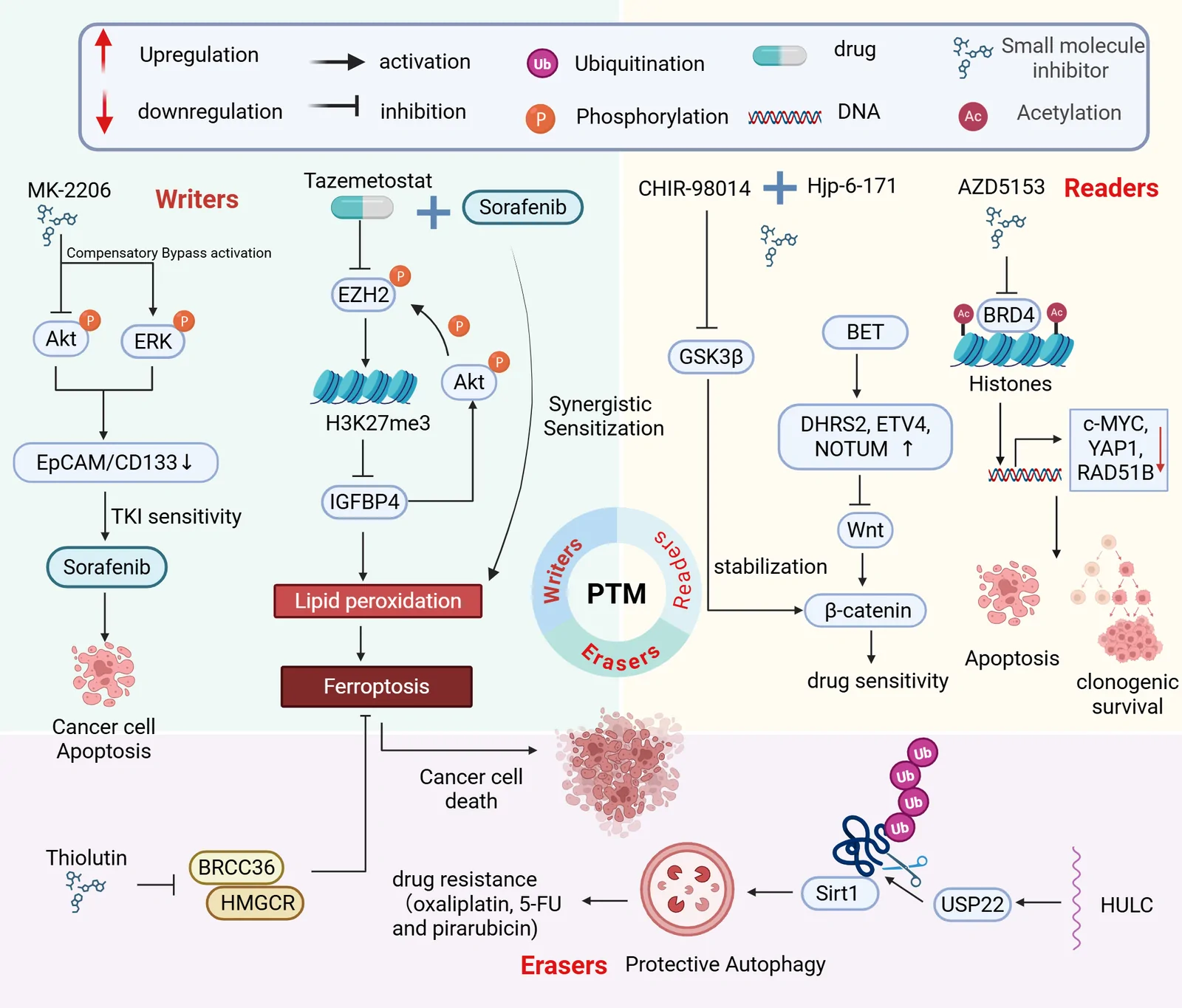
Clinical interventions targeting PTMs to regulate HCC drug resistance. Inhibitors effectively modulate the HCC resistance network by specifically intervening at three key PTM nodes. “Writers,” “Erasers,” and “Readers.” (EZH2, Enhancer of Zeste homolog 2; IGFBP4, Insulin-like growth factor-binding protein 4; GSK3β, Glycogen synthase kinase 3 beta; BRD4, Bromodomain-containing protein 4; BET, Bromodomain and Extra-Terminal motif family; DHRS2, Dehydrogenase/reductase member 2; ETV4, ETS variant transcription factor 4; NOTUM, NOTUM palmitoleoyl-protein carboxylesterase; YAP1, Yes-associated protein 1; RAD51B, RAD51 paralog B; BRCC36, BRCA1/BRCA2-containing complex subunit 36; HMGCR, 3-hydroxy-3-methylglutaryl-CoA reductase; Sirt1, Sirtuin 1; USP22, Ubiquitin-specific peptidase 22; HULC, Highly up-regulated in liver cancer).

Targeting PTM “writers” represents one of the most direct approaches to disrupting resistance-associated signaling programs. For example, the allosteric inhibitor MK-2206, targeting the core kinase AKT in the PI3K/AKT/mTOR pathway, has been reported to enhance the sensitivity of HCC cells to TKIs (118). Meanwhile, epigenetic modifying enzymes such as enhancer of zeste homolog 2 (EZH2) induce acquired resistance in HCC by mediating histone H3 lysine 27 trimethylation (H3K27me3) (119). Preclinical studies have demonstrated that the EZH2 inhibitor tazemetostat suppresses H3K27me3 deposition and reprograms the transcription of metabolism-associated genes (120). Furthermore, in sorafenib-resistant HCC models, combination treatment with tazemetostat and sorafenib enhances ferroptotic responses and restores drug sensitivity (121). Unlike inhibiting “writers,” targeting “erasers” seeks to restore tumor-suppressive PTM states or promote the degradation of oncogenic proteins. In HCC, this regulatory role of DUBs is particularly significant. Taking ubiquitin-specific peptidase 22 (USP22) as an example, it induces protective autophagy through the HULC/SIRT1 axis, directly reducing cellular sensitivity to chemotherapy (122). Another key node is BRCC36, which inhibits ferroptosis by regulating 3-hydroxy-3-methylglutaryl-CoA reductase (HMGCR) dynamics, thereby driving malignant progression, and the small molecule inhibitor thiolutin has been reported to effectively block this pathway (123). The intervention focus for “readers” has shifted to disrupting the transcriptional decoding of oncogenic signals, with current research heavily centered on the bromodomain and extraterminal domain (BET) family. The novel bivalent BRD4 inhibitor AZD5153 has demonstrated anti-HCC activity in both in vitro and in vivo models by reprogramming BRD4 chromatin occupancy and suppressing the transcription of oncogenic drivers such as c-MYC (124). Furthermore, through in-depth analysis of the BET inhibitor Hjp-6-171, genes such as dehydrogenase/reductase 2 (DHRS2) and ETS variant transcription factor 4 (ETV4) may serve as key biomarkers for predicting treatment response (125) (**Figure 3**).

Beyond conventional targeting of intracellular signaling cascades, emerging strategies aim to directly manipulate the PTM-controlled proteostasis of immune checkpoint molecules. Rather than inhibiting modifying enzymes, these approaches target checkpoint substrates themselves to restore aberrant protein turnover through PTM remodeling. PD-L1 stability is governed by an integrated network of glycosylation, ubiquitination, and phosphorylation events. Angustmycin A disrupts glycosylation-dependent stabilization of PD-L1, whereas USP47 inhibition with P5091 accelerates PD-L1 degradation by blocking deubiquitination (49, 50). CDK5 inhibition by PNU112455A prevents PD-L1 phosphorylation, disrupts PD-L1–HSC70 interaction, and redirects PD-L1 toward CMA-dependent degradation (53). In local therapy, PTM-targeting approaches exploit tumor dependence on PTM-controlled repair and immune adaptation programs. HDAC4 promotes DNA repair through chromatin remodeling, whereas HDAC4 depletion or Panobinostat treatment disrupts HDAC4 interactions with Rad51 and Ubc9, compromising homologous recombination repair (126). PARP inhibition by ABT-888 enhances radiosensitivity by interfering with poly(ADP-ribosyl)ation (127). Beyond repair inhibition, HDAC inhibitors such as AR-42 enhance radiotherapy-induced immune activation by increasing NKG2D ligand expression and promoting NK cell-mediated cytotoxicity (92, 128). Thus, PTM modulation converts local therapy from a purely cytotoxic intervention into a coordinated strategy integrating DNA damage sensitization and immune activation.

The foundation of precision therapy lies in establishing patient stratification systems based on PTM landscapes. Assessment of protein modification states may provide greater predictive value than conventional expression-based biomarkers. In the context of immunotherapy, YY1-driven GALNT16 upregulation enhances PD-L1 stability through glycosylation, and its activation status may serve as a predictor of therapeutic response (129). PTM-associated molecular signatures, including lactylation- and m6A-related gene profiles, have demonstrated prognostic stratification capacity across multiple cohorts (130). Furthermore, consensus clustering based on 21 PTM types has identified the PTM landscape as a key determinant of molecular subtypes in HCC (131).

Current translational efforts have shifted from single-node inhibition toward multimodal strategies designed to simultaneously disrupt tumor-intrinsic resistance programs and restore antitumor immunity. The oral CBL-B inhibitor NX-1607 has demonstrated T cell reinvigoration activity in early-phase clinical trials involving patients with advanced solid tumors (132). The dual PI3K/BRD4 inhibitor SF1126 has shown favorable safety profiles in a phase I clinical study of solid tumors, providing a rationale for subsequent combination with sorafenib (133). Furthermore, nanoparticle-mediated co-delivery of the SHP2 inhibitor SHP099 and the A2AR antagonist CPI-444 specifically targeting CD8^+^ T cells restored antitumor immune function by simultaneously blocking immunosuppressive signaling mediated by SHP2 and A2AR (134). Overall, the clinical translation of PTM-targeted therapies should not be viewed as the inhibition of a single modification by a single agent. Rather, it represents a multilayered strategy that simultaneously rewires resistance-associated signaling, protein stability, and transcriptional programs, thereby providing a more comprehensive approach to overcoming therapeutic resistance in HCC (**Table 3**).

**Table 3:** Pharmacological intervention strategies targeting PTM dynamics in HCC.

Type	Target	Inhibitor	Biological outcome	Reference
Writers	AKT	MK-2206	Enhances HCC cell sensitivity to Tyrosine Kinase Inhibitors (TKIs)	(118)
	EZH2	Tazemetostat	Augments ferroptosis-related effects and enhances sorafenib sensitivity	(120)
Erasers	USP22	/	Blocks protective autophagy and restores sensitivity to chemotherapeutic agents	(122)
	BRCC36	Thiolutin	Alleviates ferroptosis inhibition and suppresses HCC malignant progression	(123)
Readers	BRD4	AZD5153	Inhibits oncogene expression with potent anti-HCC activity *in vitro* and *in vivo*	(124)
	BET Family	Hjp-6-171	Regulates DHRS2 and ETV4 as biomarkers for predicting therapeutic response	(125)
PTM-regulated substrate	GMPS/PD-L1 axis	Angustmycin A	Suppresses PD-L1 expression, restores CD8^+^ T-cell function, and enhances immunotherapy sensitivity	(49)
	USP47/PD-L1 axis	P5091	Blocks PD-L1 deubiquitination, reduces PD-L1 stability, enhances anti-PD-1 therapy	(50)
	CDK5/PD-L1 axis	PNU112455A	Promotes PD-L1 degradation through T290 phosphorylation-dependent CMA pathway and enhances anti-PD-1 immunotherapy efficacy	(53)
PTM-related DNA repair	HDAC4	Panobinostat	Impairs homologous recombination repair and enhances radiation-induced cell death, increasing radiosensitivity	(126)
	PARP1	ABT-888	Enhances radiosensitivity by inhibiting PARylation	(127)

## 8 Conclusion and future perspectives

Acquired resistance in HCC cannot be attributed solely to mutations in individual target genes but rather represents a highly dynamic and multifaceted evolutionary process driven by interconnected networks of protein PTMs. In this review, we highlight the central role of PTMs in shaping therapeutic resistance across multiple treatment modalities. From activation of compensatory signaling pathways during chemotherapy and targeted therapy to the establishment of T-cell exhaustion and immune evasion within the tumor microenvironment, diverse PTMs—including phosphorylation, ubiquitination, lactylation, and glycosylation—cooperate to remodel the resistant phenotype. Through suppression of regulated cell death pathways, including apoptosis and ferroptosis, enhancement of DNA damage repair, and maintenance of cancer stem cell-associated epigenetic programs, these PTM-dependent events collectively reinforce tumor adaptation and therapeutic resistance in HCC.

In response to the complexity of PTM-driven resistance networks, therapeutic strategies are increasingly shifting from conventional single-kinase inhibition toward integrated modulation of the PTM “writer–eraser–reader” axis. Notably, the diversity and interconnectedness of PTM networks provide opportunities for the development of multi-target therapeutic approaches. In this context, natural small-molecule compounds with pleiotropic regulatory activities, particularly bioactive constituents derived from traditional Chinese medicine, represent a valuable source of potential sensitizing agents with favorable efficacy and safety profiles. Rational integration of these compounds with existing immunotherapies or targeted therapies may offer a promising strategy for overcoming therapeutic resistance in HCC.

Despite the translational potential of PTM-based strategies for overcoming therapeutic resistance, several challenges continue to hinder their clinical implementation. At the technological level, current PTM profiling platforms remain constrained by limited yield, insufficient purity, and inadequate analytical sensitivity, restricting comprehensive coverage, quantitative reproducibility, and dynamic monitoring of PTM landscapes. At the therapeutic level, existing PTM-targeting inhibitors and PROTAC-based approaches often lack sufficient tissue selectivity, resulting in potential off-target toxicity. At the validation level, most discoveries remain confined to cell models and xenograft systems, with limited confirmation in large-scale clinical cohorts. Mechanistically, the dynamic crosstalk among different PTM types and its contribution to resistance evolution remain poorly defined. Furthermore, the heterogeneity of PTM regulatory networks across distinct HCC etiological backgrounds and their impact on treatment adaptation remain largely unexplored, limiting the precision application of PTM-based biomarkers and therapeutic strategies.

Future studies should integrate single-cell proteomics with spatial transcriptomics to define spatiotemporal PTM landscapes and employ CRISPR screening to identify key modification enzymes driving cross-resistance. Microenvironment-responsive nanocarriers and targeted protein degraders, including PROTACs and molecular glues, may facilitate tumor-selective intervention and warrant further validation in PDX models. Clinically, PTM-based liquid biopsy panels combined with etiological and molecular stratification may enable personalized combination strategies. Ultimately, multidisciplinary approaches will be essential for translating PTM biology into improved therapeutic outcomes for patients with HCC.

## Glossary

5-FU: 5-Fluorouracil
A2AR: A2A receptor
ACLY: ATP citrate lyase
ACVR2A: Activin A receptor type 2A
Acetyl-CoA: Acetyl coenzyme A
AK2: Adenylate kinase 2
AKT: Ataxia telangiectasia mutated
AMPK: AMP-activated protein kinase
ATM: Ataxia telangiectasia mutated
BCL-2: B-cell lymphoma 2
BET: Bromodomain and extraterminal domain
β-catenin: Catenin beta 1
β-TrCP: Beta-transducin repeat-containing protein
BRCA1: Breast cancer susceptibility gene 1
BRD4: Bromodomain-containing protein 4
CBL-B: Casitas B-lineage lymphoma proto-oncogene B
CCT3: Chaperonin-containing TCP1 subunit 3
CD36: Cluster of differentiation 36
CDK5: Cyclin-dependent kinase 5
CDK11B: Cyclin-dependent kinase 11B
CHK2: Checkpoint kinase 2
CMA: Chaperone-mediated autophagy
CREB: cAMP response element-binding protein
CRTC2: CREB-regulated transcription coactivator 2
CSCs: Cancer stem cells
CSNK1A1: Casein kinase 1 alpha 1
c-Myc: MYC proto-oncogene protein
DDR: DNA damage repair
DDOST: Dolichyl-diphosphooligosaccharide—protein glycosyltransferase non-catalytic subunit
DHODH: Dihydroorotate dehydrogenase
DHRS2: Dehydrogenase/reductase 2
DUBs: Deubiquitinating enzymes
DTX2: Deltex E3 ubiquitin ligase 2
EGFR: Epidermal growth factor receptor
EMT: Epithelial–mesenchymal transition
ERK: Extracellular signal-regulated kinase
ETV4: ETS variant transcription factor 4
EZH2: Enhancer of zeste homolog 2
FASN: Fatty acid synthase
FOXP3: Forkhead box P3
FOXK2: Forkhead box K2
FSP1: Ferroptosis suppressor protein 1
GLS1: Glutaminase 1
GMPS: Guanosine monophosphate synthetase
GPX4: Glutathione peroxidase 4
GSK-3β: Glycogen synthase kinase 3 beta
H3K27ac: Histone H3 lysine 27 acetylation
H3K27me3: Histone H3 lysine 27 trimethylation
HCC: Hepatocellular carcinoma
HDAC2: Histone deacetylase 2
HELLS: Lymphoid-specific helicase
HIF-1α: Hypoxia-inducible factor 1-alpha
HMGCR: 3-Hydroxy-3-methylglutaryl-CoA reductase
HR: Homologous recombination
HSC70: Heat shock cognate protein 70
HSD17B4: Hydroxysteroid 17-beta dehydrogenase 4
ICIs: Immune checkpoint inhibitors
IFN-γ: Interferon-gamma
IGF2BP3: Insulin-like growth factor 2 mRNA-binding protein 3
ISG15: Interferon-stimulated gene 15
ITGB5: Integrin beta 5
JNK: c-Jun N-terminal kinase
KAT2A: Lysine acetyltransferase 2A
KAT8: Lysine acetyltransferase 8
KEAP1: Kelch-like ECH-associated protein 1
LGALS3: Galectin-3
LKB1: Liver kinase B1
LOXL3: Lysyl oxidase-like protein 3
LTβR: Lymphotoxin-β
mRNA: Messenger RNA
MRE11: Meiotic recombination 11 homolog 1
mTOR: Mechanistic target of rapamycin
MVP: Major vault protein
NANOG: Homeobox protein NANOG
NAT10: N-acetyltransferase 10
NCOA4: Nuclear receptor coactivator 4
NEDD4: Neural precursor cell expressed developmentally down-regulated protein 4
NF-κB: Nuclear factor kappa B
NHEJ: Non-homologous end joining
NONO: Non-POU domain-containing octamer-binding protein
NOTCH1: Notch receptor 1
NRF2: Nuclear factor erythroid 2-related factor 2
NPM1: Nucleophosmin 1
NUPR1: Nuclear protein 1
O-GlcNAc: O-linked N-acetylglucosamine
OGT: O-GlcNAc transferase
OTUB1: OTU deubiquitinase, ubiquitin aldehyde-binding 1
PARK7: Parkinson disease protein 7
PARP: Poly(ADP-ribose) polymerase
PCK: Phosphoenolpyruvate carboxykinase
PD-L1: Programmed death-ligand 1
PI3K: Phosphatidylinositol 3-kinase
PKM2: Pyruvate kinase M2
PLEKHG5: Pleckstrin homology and RhoGEF domain-containing family G member 5
PP2A-B55β: Protein phosphatase 2A regulatory subunit B55β
PRDX1: Peroxiredoxin 1
PRMT3: Protein arginine methyltransferase 3
PRMT5: Protein arginine methyltransferase 5
PROTAC: Proteolysis-targeting chimera
PTEN: Phosphatase and tensin homolog
PTMs: Protein post-translational modifications
p300: E1A-binding protein p300
Rac1: Ras-related C3 botulinum toxin substrate 1
RBBP6: Retinoblastoma-binding protein 6
RBPJ: Recombination signal-binding protein for immunoglobulin kappa J region
RNF135: Ring finger protein 135
ROS: Reactive oxygen species
RPL14: Ribosomal protein L14
S100A14: S100 calcium-binding protein A14
SENP3: Sentrin/SUMO-specific protease 3
SETMAR: SET domain and mariner transposase fusion gene
SHP-1: Src homology 2 domain-containing protein tyrosine phosphatase 1
SHP2: Src homology region 2 domain-containing phosphatase-2
SIRT1: Silent information regulator 1
SLC7A11: Solute carrier family 7 member 11
SMC4: Structural maintenance of chromosomes 4
SRSF11: Serine/arginine-rich splicing factor 11
STAT3: Signal transducer and activator of transcription 3
STING: Stimulator of interferon genes
STT3A: STT3 oligosaccharyltransferase complex catalytic subunit A
SUMO: Small ubiquitin-like modifier
TAMs: Tumor-associated macrophages
TCR: T-cell receptor
TFRC: Transferrin receptor 1
TKT: Transketolase
TME: Tumor microenvironment
TRAIL: Tumor necrosis factor-related apoptosis-inducing ligand
Treg: Regulatory T cells
UFM1: UFM1 modifier 1
USP1: Ubiquitin-specific peptidase 1
xCT: xCT cystine/glutamate antiporter
XPO1: Exportin 1
YIPF5: Yip1 domain family member 5
ZNF207: Zinc finger protein 207
