# Correction: Post-translational modification in hepatocellular carcinoma resistance: molecular mechanisms and therapeutic targeting

**DOI:** 10.3389/fimmu.2026.1986779

**Published:** 2026-09-18

**Authors:** Simin Feng, Jie Hu, Xiaoxi Guo, Ziyi Xu, Hailiang Wang, Huanzhang Niu, Xinyang Li

**Affiliations:** 1 Department of Interventional Radiology, The First Affiliated Hospital, College of Clinical Medicine, Henan University of Science and Technology, Luoyang, China; 2 Department of Anesthesiology, Luoyang Central Hospital, Luoyang, China; 3 Henan Key Laboratory of Cancer Epigenetics, Cancer Institute, The First Affiliated Hospital, College of Clinical Medicine, Henan University of Science and Technology, Luoyang, China; 4 Department of Gastrointestinal Surgery, People’s Hospital of Zhengzhou University, Henan Provincial People’s Hospital, Zhengzhou, China

**Keywords:** hepatocellular carcinoma, immunotherapy, post-translational modifications, targeted therapy, therapeutic resistance

There was a mistake in **Tables 1**, **2** as published. In both tables, the latter part of the content was inadvertently duplicated from the earlier content within the same tables. The corrected **Tables 1**, **2** appear below, with all duplicate rows removed. The rest of the data in the tables are correct and remain unchanged.

**Table 1:** PTMs in HCC Drug Resistance.

Drug	Target	PTM type	Modifying enzyme/regulatory factor	Mechanism	Intervention strategy	Reference
Oxaliplatin	LOXL3 (S704)	Phosphorylation	AK2	AK2/LOXL3/DHODH/ferroptosis/resistance	Leflunomide	(15)
	IGF2BP1 (R452)	Methylation	PRMT3	PRMT3/IGF2BP1/HEG1/survival signaling/resistance	PRMT3 as biomarker	(16)
5-FU	SMC4	Deubiquitination	USP39	USP39/SMC4/cell cycle/proliferation/resistance	SMC4/USP39 knockdown	(19)
	YIPF5	Ubiquitination	(Mitophagy)	YIPF5/mitophagy/senescence/5-FU sensitivity	Salidroside	(20)
FOLFOX	HELLS	Deubiquitination & SUMOylation	USP1	USP1/HELLS/EMT/HRR/chemoresistance	ML323	(17)
	Histone H3 (K14)	Lactylation	p300 (EP300)	p300/H3K14la/NEDD4/PTEN/AKT/resistance	Glycolysis inhibitors	(21)
	IGF2BP3 (K76)	Lactylation	PARK7	PARK7/IGF2BP3/FSP1/ferroptosis/resistance	Blocking antibody	(22)
Sorafenib	PCK1 (K473)/PCK2 (K491)	Acetylation	KAT8	KAT8/PCK1/PCK2/metabolic transition/resistance	PCK2 inhibition	(23)
	GLS (Y308/S314)	Phosphorylation & Ubiquitination	S100A14	S100A14/GLS/oxidative stress/apoptosis/resistance	GLS inhibition	(24)
	SIRT1/AMPK	Deacetylation & Phosphorylation	SIRT1	NAD/SIRT1/AMPK/mTOR/apoptosis/resistance	SIRT1 inhibition	(25)
	NPM1 (K54)	Acetylation	XPO1	XPO1/NPM1/Vimentin/EMT/resistance	KPT-8602	(26)
	PLEKHG5	Deacetylation	HDAC2	HDAC2/PLEKHG5/Rac1/AKT/NF-kB/resistance	HDAC2 inhibitors	(27)
	ITGB5/EGFR	Phosphorylation (ITGB5)	CSNK1A1/EPS15	ITGB5/EPS15/EGFR/CSNK1A1/AKT/mTOR/resistance	Targeting ITGB5/CSNK1A1 axis	(28)
	CHK2/BRCA1	Phosphorylation	NOTCH1/ATM axis	NOTCH1/ATM/CHK2/BRCA1/genomic stability/resistance	ATM inhibition	(29)
	CDK11B/H2B (K120)	Deubiquitination	USP22	USP22/CDK11B/H2B/TFRC/ferroptosis/resistance	Hyperoside	(30)
	NCOA4	DeISGylation	USP18	USP18/NCOA4/ferroptosis/acquired resistance	Hyperoside	(31)
	CCT3 (K21)	K6-linked Deubiquitination	USP11	USP11/CCT3/ACTN4/TFRC/ferroptosis/resistance	CCT3/USP11 inhibition	(32)
	GPX4 (S2)	Dephosphorylation	PP2A-B55β	PP2A-B55β/GPX4/ferroptosis/resistance	LB-100 (PP2A inhibitor)	(33)
Lenvatinib	EGFR	N-glycosylation	DDOST	DDOST/EGFR/survival signaling/acquired resistance	DDOST inhibition	(38)
	CRTC2	Dephosphorylation	PP2A	PP2A/CRTC2/CREB/survival genes/resistance	CRTC2 inhibition	(39)
	LGALS3	Deubiquitination	USP15	USP15/LGALS3/AKT/mTOR/resistance	LGALS3/USP15 inhibition	(40)
	HSD17B4	Ubiquitination (Degradative)	DTX2	DTX2/HSD17B4/lipid metabolism/ferroptosis/resistance	DTX2 inhibition	(41)
Regorafenib	RPL14 (R141)	Symmetric dimethylation	PRMT5	PRMT5/RPL14/DNA damage/resistance	PRMT5 inhibition	(42)
Multikinase Inhibitors	FOXK2/SLC7A11	O-GlcNAcylation	OGT	ROS/OGT/FOXK2/SLC7A11/ferroptosis/resistance	OGT inhibitors	(43)
	PRDX1 (K67)/NRF2	Lactylation	ZNF207	ZNF207/PRDX1/NRF2/antioxidant/ferroptosis/resistance	NRF2 inhibition	(44)
Immunotherapy	PD-L1	N-glycosylation	STT3A/GMPS	GMPS/STT3A/PD-L1/CD8+ T cell/immune evasion	Angustmycin A	(49)
	PD-L1	Deubiquitination	USP47	USP47/PD-L1/protein stability/immune evasion	P5091	(50)
	PD-L1/MVP	Lactylation & Ubiquitination	MVP/beta-TrCP	Lactylation/MVP/PD-L1/beta-TrCP/immune evasion	Lactylation inhibitors	(51)
	PD-L1 (T290)	Phosphorylation	CDK5/HSC70	CDK5/PD-L1/T290/HSC70/CMA/immune evasion	PNU112455A	(53)
	TIM-3 (C296)	Palmitoylation	DHHC9/HRD1	DHHC9/TIM-3/HRD1/immune evasion/resistance	DHHC9 knockdown	(55)
	SHP-1	Dephosphorylation	SHP-1	SHP-1/TCR signaling/HMGCR/T-cell exhaustion	SHP-1-KO T cells	(60)
	CD36/RBPJ	N-glycosylation/UFMylation	USP7/AMPK	CD36/AMPK/USP7/RBPJ/T-cell exhaustion/evasion	Targeting USP7/RBPJ	(61)
	FASN/STAT3	Palmitoylation	Riplet/RNF135	Riplet/FASN/palmitic acid/STAT3/T-cell exhaustion	FASN inhibitors	(62)
	STAT3 (Y705)	Dephosphorylation	SHP-2/TIGIT	TIGIT/SHP-2/STAT3/GLUT1/glycolysis/resistance	anti-TIGIT/SHP099/JSI-124	(63)
	H3K18la (TAMs)/NUPR1	Lactylation	Lactate (Tumor-derived)	Lactate/H3K18la/NUPR1/M2 polarization/evasion	Targeting NUPR1	(66)
	STAT3	Phosphorylation	IL-6/SDF-1α	TAFs/SDF-1α/CXCR4/IL-6/STAT3/MDSC induction/immune suppression	SDF-1α inhibition	(68)
	STAT3 (Y705)	Phosphorylation	CCRK	CCRK/STAT3/E4BP4/IL-10/IL-12/immune suppression	Stattic/Ccrk-knockdown	(70)
	SMAD2/3/Foxp3	Phosphorylation & Lactylation	ACVR2A/Lactate	ACVR2A/SMAD/MCT4/lactate/Foxp3/Treg/evasion	MCT4 inhibitors	(71)
	LTβR/RORC/Foxp3	N-glycosylation & Ubiquitination	PELI1/SMURF1	LTβR/PELI1/RORC/Foxp3/Th17/Treg/ratio/evasion	LTβR-CAR-T + 2-DG	(73)
Lenvatinib (TKI)	Histone H3 (K18la)	Lactylation	BRPF1	GPX8/lactate/MCT1/H3K18la/BRPF1/H3K14ac/EGFR/resistance	AZD3965; GSK5959	(76)
Sorafenib/Lenvatinib (TKIs)	Histone H4 (K12la)	Lactylation	AARS1/HDAC11	AARS1/H4K12la/RAPGEF3/RAP1/resistance	ESI-09	(77)
Anti-PD-1 (ICI)	PGAM1 (K251)	Ubiquitination & Lactylation	JOSD1/AARS1	JOSD1/AARS1/PGAM1/PTM crosstalk/glycolysis/immune suppression	JOSD1 inhibition + anti-PD-1 therapy	(78)
Anti-PD-1/PD-L1 (ICI)	ATF6 (K424la)	Lactylation	AARS1	AARS1/ATF6/K424la/TDO2/kynurenine/Treg/glycolysis/immune resistance	β-alanine+ PD-1/PD-L1 blockade	(79)
Radiotherapy (RT)	TKT	PARylation	PARP1	PARP1/TKT/HR-NHEJ/DNA repair/radioresistance	TKT depletion or PARP1 inhibition	(83)
	Ku complex	PARylation	PC4/PARP1	PC4/PARP1/Ku complex/NHEJ/DNA repair/radioresistance	PC4 targeting	(84)
	NONO (S147)	O-GlcNAcylation	NONO/SFPQ/SETMAR	NONO/SETMAR splicing/Ku70/NHEJ/DNA repair/radioresistance	Targeting NONO O-GlcNAcylation	(85)
	SRSF11 (K419)	Succinylation	KAT2A	KAT2A/SRSF11/RAD52 splicing/RAD51-HR/DNA repair/radioresistance	KAT2A-SRSF11 axis disruption	(86)
	SLC27A5	Ubiquitination	UBAP2	UBAP2/SLC27A5/RAD51/HR/DNA repair/radioresistance	Targeting UBAP2/SLC27A5 axis	(87)
TACE	c-Raf (S338)/c-Myc (S62)	Phosphorylation	TKT/RAF1	TKT/RAF1/ERK/c-Myc/stability/proliferation/TACE resistance	Targeting TKT/c-Myc axis	(89)
	ITGB3	Ubiquitination	SERPINA1/ITCH	SERPINA1/ITGB3/ITCH/proteasomal degradation/oncogenic signaling/TACE resistance	Targeting SERPINA1/ITGB3 axis	(90)
	G6PD (T236/K238)	Phosphorylation & SUMOylation	PKCδ/SENP1	PKCδ/G6PD/PPP/redox balance/survival/TACE resistance	PKCδ inhibition	(91)
Radiotherapy (immune modulation)	PALB2 (K628)/Histone H3 (K4)	Acetylation	MORF4L1	MORF4L1/PALB2/H3K4/DNA repair/immune response/radioresistance	Argatroban + RT	(93)
TACE (immune modulation)	Macrophage/SPP1+ macrophages	Lactylation	Lactate metabolism	Lactate/SPP1+ macrophage/CD8+ T cell dysfunction/immune suppression/TACE resistance	Targeting lactate-induced lactylation or SPP1-CD44 axis	(94)

**Table 2:** PTM-mediated molecular events driving HCC persistence and recurrence.

PTM type	Target molecule	Upstream	Mechanism	Biological outcome	Reference
Phosphorylation	PKM2 (S37)/Histone H3 (T11)	EGFR -ERK	EGFR/PKM2/H3-T11/chromatin remodeling	Metabolic-epigenetic adaptation and subclonal persistence under therapeutic stress	(96)
	p53 (S46)	ZNF498	ZNF498/p53/S46 phosphorylation/apoptosis/ferroptosis	Survival advantage; tumor persistence	(107)
	c-Myc (S62)	TKT	TKT/RAF1/ERK/c-Myc	c-Myc stability; stemness maintenance	(108)
	c-Myc (S62/T58)	TTC36	TTC36/PP2A/GSK3b/c-Myc	c-Myc stabilization; CSC maintenance and survival advantage	(109)
	β-catenin (S33/S37/T41/S45)	GSK-3β	GSK-3β/β-catenin phosphorylation/Wnt signaling	β-catenin stabilization; HCC progression	(109)
	YAP/TAZ	Hippo pathway (MST1/2)	Hippo/YAP/TAZ/Wnt signaling	YAP/TAZ activation; tumor cell plasticity	(113)
	ERK/p38 MAPK	miR-122	miR-122/p38 MAPK/ERK phosphorylation balance	Low p-ERK/p-p38 ratio induces tumor dormancy program	(114)
	STAT3/P70S6K/AMPKα	CAV1	CAV1/STAT3/P70S6K/AMPKα phosphorylation signaling	Dormancy maintenance; recurrence-associated cell survival	(115)
Ubiquitination	KEAP1/NRF2	HECTD2	HECTD2/KEAP1/NRF2/redox adaptation/resistance	Oxidative stress tolerance; resistant subclone survival	(102)
Deubiquitination	ACLY(K726)	HIF-1α/USP13	HIF-1a/USP13/ACLY/stability/metabolic adaptation	Hypoxia adaptation and survival advantageDrives ferroptosis resistance and tumor immune evasion	(98)
	FKBP12	USP22	USP22/FKBP12/mTORC1/autophagy	Promotes HCC tumorigenesis and stemness	(110)
	c-Myc	WDR20/USP12/46	WDR20/USP12/46/c-Myc deubiquitination/stability	Senescence avoidance; HCC cell survival maintenance	(116)
Acetylation	ACLY (K468)	NAT10	NAT10/ACLY/H3K27ac/nuclear acetyl-CoA	Metabolic-epigenetic remodeling and chemoresistant clone persistence	(99)
	p53 (K382)	CBP/p300	p53/K382 acetylation/apoptotic transcription	Apoptosis induction; stress response regulation	(1)
Histone Lactylation	HECTD2 (Promoter)	Lactate	Lactate/H3K18la/HECTD2/NRF2	Upregulates HECTD2; drives lenvatinib resistance	(102)

The original version of this article has been updated.

